# Open consent, biobanking and data protection law: can open consent be ‘informed’ under the forthcoming data protection regulation?

**DOI:** 10.1186/s40504-014-0020-9

**Published:** 2015-01-24

**Authors:** Dara Hallinan, Michael Friedewald

**Affiliations:** Competence Center Emerging Technologies, Fraunhofer Institute for Systems and Innovation Research ISI, Breslauer Strasse 48, 76139 Karlsruhe, Germany

**Keywords:** Genetics, Genomics, Data protection, Data protection regulation, Consent, Open consent, Biobanks

## Abstract

This article focuses on whether a certain form of consent used by biobanks – open consent – is compatible with the Proposed Data Protection Regulation. In an open consent procedure, the biobank requests consent once from the data subject for all future research uses of genetic material and data. However, as biobanks process personal data, they must comply with data protection law. Data protection law is currently undergoing reform. The Proposed Data Protection Regulation is the culmination of this reform and, if voted into law, will constitute a new legal framework for biobanking. The Regulation puts strict conditions on consent – in particular relating to information which must be given to the data subject. It seems clear that open consent cannot meet these requirements. 4 categories of information cannot be provided with adequate specificity: purpose, recipient, possible third country transfers, data collected. However, whilst open consent cannot meet the formal requirements laid out by the Regulation, this is not to say that these requirements are substantially undebateable. Two arguments could be put forward suggesting the applicable consent requirements should be rethought. First, from policy documents regarding the drafting process, it seems that the informational requirements in the Regulation are so strict in order to protect the data subject from risks inherent in the use of the consent mechanism in a certain context – exemplified by the online context. There are substantial differences between this context and the biobanking context. Arguably, a consent transaction in the biobanking does not present the same type of risk to the data subject. If the risks are different, then perhaps there are also grounds for a reconsideration of consent requirements? Second, an argument can be made that the legislator drafted the Regulation based on certain assumptions as to the nature of ‘data’. The authors argue that these assumptions are difficult to apply to genetic data and accordingly a different approach to consent might be preferable. Such an approach might be more open consent friendly.

## Introduction

More and more biobanks are being set up to facilitate wide ranging, population based genomics research. Such biobanks act as research infrastructure – collecting data and samples from research subjects and making this material available, on application, to researchers. The scope and accuracy of genomic research depends on the size of data sets and the numbers of samples available to researchers who apply. Accordingly, in order to maximize the utility of samples and data collected in biobanks, there should be as few limits on their use as possible. Accordingly, many large scale biobanks collect materials prospectively – they do not state the type of research the materials will be used for at the moment of collection.

In order to conduct medical research on competent adults, researchers have generally been obliged to seek consent before conducting their work. Prior to biobanking, such consent was only regarded as legitimate under certain conditions. These required the research subject to be informed, in advance, as to the specific research which would be conducted. Prospective collection as occurs in the biobanking context is not possible under traditional models of consent. Accordingly, in order to bridge this impasse, alternative consent models were proposed for biobanking research. One such model is that of open consent. In open consent, the participant need only engage with the biobank once. The consent given in this engagement is valid for the extraction of the sample, the storage of the sample and data, and all further research uses of collected materials.

As biobanks process ‘personal data’, European data protection law is applicable to their operation. Despite the clarity that data protection law applies, there are a number of uncertainties as to the precise application of the current piece of overarching legislation – Directive 95/46 (European Parliament & European Council 1995). Whether open consent is legitimate is one such uncertainty.^a^ In particular, the Directive requires that any consent be ‘specific’ and ‘informed’. It seems that biobanks operating with open consent cannot meet these obligations.

However, European data protection law is currently under review. This review has resulted in a Proposed Data Protection Regulation. This Regulation will replace the Directive and will provide a new background to the relationship between biobanks and data protection

This article considers the status of open consent in light of the Regulation.^b^ A number of questions arise: Must biobanks legitimate processing through consent under the Regulation, or are there other possibilities? Which information, and of which specificity, does the Regulation oblige the biobank to provide to the data subject? Can this be provided under an open consent procedure? If open consent cannot find legitimation under the Regulation as it is, what are its prospects moving forward?

The article can be read in 4 parts:

Part 1 describes the open consent process and the obligations laid out by the Regulation relating to biobanks using open consent. It begins by explaining how open consent works and what differentiates it from other modes of consent (section 1). It proceeds by explaining the function of the Regulation, and by explaining its application to biobanks – concluding that the Regulation applies to both samples and data, and continues to apply until they are destroyed, and that biobanks can always be considered to process sensitive personal data (section 2). When sensitive data are processed, this must be justified under one of the grounds in Article 9. The article argues that, while there are a number of grounds which could apply, the most relevant is consent (section 3). The legitimation to process sensitive data following the data subject’s consent is provided by Article 9(2)(a) and can only succeed under a number of conditions – including that consent is ‘specific and informed’. The article then proceeds to elaborate what is meant by ‘specific and informed’ under the Regulation. It explains that certain categories of information must be provided to the data subject by the biobank (section 4), and that this information must be of a certain specificity (section 5).

Part 2 considers whether the open consent process can fulfil the informational obligations laid out in sections 4 and 5. The article suggests that the open consent process cannot provide the relevant specificity of information in relation to 4 categories of information; purpose of processing (section 6); recipients of processing (section 7); the third countries to which data will be transferred (section 8); the type of data which will be processed (section 9). With this analysis, the article concludes that, against the normal framework of informational obligations laid out be the Regulation, open consent cannot succeed.

Part 3 then considers arguments which suggest that the normal framework provided by the Regulation ought not apply. Such arguments suggest that, under the law in the Regulation, open consent could still count as ‘informed and specific’ as there are mechanisms which allow a relaxation of the problematic consent conditions. The first argument suggests that the expectations of the parties to the consent transaction have a performative effect on relevant informational obligations (section 10). The second argument suggests that, as biobanks employ other mechanisms of privacy protection – ‘buffer safeguards’ – these might provide reason for the problematic informational obligations to be relaxed (section 11). The article finds some justification for these arguments in the law. However, such justification is inadequate for open consent to succeed.

Part 4 finally considers a different form of argument supporting open consent. This form of argument suggests the logic behind the conditions laid down by the Regulation does not apply to biobanking. Accordingly, an argument can be made that the applicable conditions should be reconsidered. The article begins this section with an introduction to the concepts behind this form of argument – including pointing out that the conditions of consent are not rights, but are ties to assumptions as to the substance of regulation, the function of consent, and the actors involved (section 12). Two different arguments are put forward. The first argument holds that the specificity of the informational obligations in the Regulation was set with a particular processing context in mind. In this context there was a need to protect the data subject from particular risks in of the consent procedure. The argument suggests that such risks are not nearly so pronounced in the biobanking context and therefore the justification behind the high specificity threshold is limited (section 13). The second argument observes that the Regulation was drafted with a specific concept of ‘data’ in mind. Based on this concept, the legislator has constructed a concept of consent which requires a specific description of the data which have been collected. However, genetic data is better understood as a future source of data, than as data itself. This means that any concept of consent which focuses on defining ‘data’ which have been collected will be of limited applicability (section 14).

In summary, there seems no way open consent can meet the requirement under the Regulation for consent to be ‘specific and informed’. However, there are strong arguments suggesting that the Regulation’s conditions are poorly suited to the biobanking context and to the processing of genetic data. In this regard, the authors feel there should be a political discussion about whether to provide a specific set of consent conditions for biobanking.

### Open consent

The site of medical research has traditionally been the human body – whether alive or dead. Modern medical ethics emerged with this concept of research as its template (with the focus of ethical consideration naturally being on research done on living subjects). Such research is characterized by certain features. First, it engages directly with the research subject’s physical person. The risks associated with this form of research are therefore predominantly physical. Second, in each instance invasive research occurred, the purpose of the invasive procedure could be clarified in advance. Conditions for a legitimate consent in medical ethics reflected these characteristics. In particular, as part of any legitimate consent, specific information as to the nature, form and risks of the particular proposed research had to be communicated to the participant in advance, before any research could go ahead (Biggs 2009, pp 17–35).

Genetics, and later genomics opened up new possibilities in how to conduct research. Importantly, genetic research can be conducted on extracted samples, rather than on the research subject’s physical person. In essence, no human subject is required for the act of research (although the sample must still be extracted). However, genetics and genomics research require something else – human samples and data. The scope, quality and effectiveness of such research is predicated on the number of samples and quantity of data used. The more relevant genetic information (and often health and environmental information – information relating to subject lifestyle etc.) used in any study, the more reliable the results of that study will be.^c^ Equally, the more data and samples that are available for reuse, the more total studies that can be conducted and the more studies can be verified for accuracy (Kaye 2012, pp 32–45).

Biobanks – stores of genetic material and information, which can then be used in research projects – form a key part of the infrastructure supporting genetics and genomics research. As the importance of samples and data has grown in research, and as genomics research attempts to analyse ever expanding data sets, biobanks are becoming larger and more prospective – collecting samples with no particular research purpose in mind (Expert Group on Dealing with Ethical and Regulatory Challenges of International Biobank Research (2013)).

However, the stringent conditions of the traditional concept of consent in medical research stand in direct contradiction to the prospective collection approach – the requirement to specify specific research in advance standing in the way of extracting maximum research utility from collected samples.

In response to this obstruction, arguments were put forward suggesting that biobanking was a novel research context. Such arguments agreed that a respect for proband autonomy and self-determination remained an important ethical principle in biobank research, but suggested that the traditional concept of consent was unnecessarily restrictive. On the one hand, it was argued that biobanking held great promise for health research and that maintaining strict consent requirements would hinder the realisation of this promise (Hansson et al. 2006, p 267). On the other hand, it was argued that that the form of physical risk inherent in invasive research is different to (the arguments often suggest that it is also greater than) the informational risk present in biobanking research (Mascalzoni et al. 2008). On the back of these arguments, a number of novel consent modes were proposed as more suitable alternatives for biobanking. For example:

Democratic community consent (also known as presumed consent or opt-out), was employed in Iceland to legitimate the collection and use of the Icelandic population’s samples and data (Árnason and Árnason 2004, pp 164–177). This approach assumed every individual’s consent to the future use of their samples and materials. The individual was not specifically asked for consent at any point, but was given the option to opt out of the project^d^.

Dynamic consent imagines a ‘personalised, digital communication interface’ which allows biobank/researchers to establish a continuous communication with research subjects (Kaye et al. 2014, pp 1). Research subjects can be presented with specific projects and information and, in turn, continuously tailor their consent preferences according to their desires and needs^e^.

Sectoral consent imagines a limited extension to the boundaries of specific consent. Whilst specific consent demands that the specifics of the project are given to the research subject in advance, sectoral consent allows a research subject to consent to research in a given area – for example, a data subject would be allowed to consent to all cancer research (American Society of Human Genetics, 1996)^f^.

However, of all the proposed novel forms of consent, it is ‘open consent’ – also known as ‘broad consent’, ‘general consent’ and even ‘blanket consent’ – which strays furthest from the traditional model of specific informed consent^g^. Open consent has a history stretching back over a decade (Chadwick and Berg 2001). In this time, multiple definitions have been proposed, for example: Chadwick and Berg (2001), p 320 suggest that: ‘consent could be introduced, such as consent to entry into a sample collection…and to further (more general) research’. ‘Lunshof et al. (2008), p 409 suggest – in relation to the Personal Genome Project – that ‘open consent means that volunteers consent to unrestricted [research] re-disclosure of data originating from a confidential relationship, namely their health records, and to unrestricted [research] disclosure of information that emerges from any future research on their genotype-phenotype data set, the information content of which cannot be predicted’; Nomper (2005), p 83 suggests – in relation to population biobanks – that: ‘Open consent is the research subject’s affirmative agreement to participate in a population genetic database and in research projects that use tissue and data from that database’. Whilst there are minor differences between definitions – and this suggests the concept is still in definitional flux – there are certain core features of open consent which are common across all definitions. These characterise the concept and to differentiate it from all other forms of consent.

1. The research subject actively gives consent (unlike presumed consent) only once to the biobank (unlike dynamic consent).
2. The research subject is not asked to give consent to a specific research project (unlike traditional specific informed consent) or to an area of research (unlike sectoral consent). The subject gives consent to the use of their sample and data for all future research purposes. At the moment of collection, the research projects – nor even research areas – in which tissue samples and data will be used, are not precisely defined.
3. In most biobanks operating open consent, researchers wishing to conduct research on samples will apply to the biobank for the opportunity to use stored tissue and data.^h^ The biobank (or more precisely, the governance systems of the biobank) will then decide whether a research project will be permitted to use the requested material. The research subject plays no role in this decision.

Open consent is also the most prominently used of the novel forms of consent. It is not hard to see why. On the one hand, it allows the maximum research utility to be extracted from each sample – the consent includes no limits to the scope of research uses to which the sample could be put. On the other hand, the consent procedure demands minimal organisational and administrative effort – one meeting with the subject, one form signed and the consent procedure is concluded. Open consent has proven particularly popular with population biobank projects. The donor consent form for the Estonian Geenivaramu states in Article 10: ‘By signing this document, I give my free and informed consent to… 4) Have the tissue sample, description of my state of health and my genealogy entered in the Gene Bank in coded form; 5) The use thereof for genetic research, public health research and statistical and other purposes in accordance with the law’ (TÜ Eesti Geenivaramu 2007). The donor consent form for the UK biobank states: ‘I give permission for long-term storage and use of my blood and urine samples for health-related research purposes (even after my incapacity or death)’ (UK Biobank 2013).

### The data protection regulation and its application to biobanking

Since 1995, the key legal instrument elaborating European data protection law has been Directive 95/46 (European Parliament & European Council 1995). However, the role of the Directive in the regulation of biobanks was highly uncertain. On the one hand, the Directive was not designed with biobanks in mind, and it was therefore unclear as to how and when its provisions should be applied. On the other hand, in the absence of European level clarity on the application of the Directive, Member States provided local approaches, which in turn led to a fragmentation of legal approaches across Europe (Schulte in den Bäumen et al. 2010, pp 36).

In the 18 years since the drafting of the Directive, much has changed^i^. Accordingly, in 2009 the European Commission began an investigation into the reform of data protection law. The reform process took a big step forward in January 2012, when the Commission released a draft Data Protection Regulation proposed as a replacement to Directive 95/46 (European Commission 2012a). This Regulation has since been debated in the Parliament, which, earlier in 2014 approved an amended ‘Consolidated Regulation’ for consideration before the Council (The European Parliament (2013)). The Council have also suggested a number of further amendments to the Commission’s original version. (The Council of the European Union (2013)). If adopted, this Regulation will become the central instrument in European data protection law. As opposed to Directives – which lay out principles to be transposed into Member State law – Regulations are directly applicable as law in each Member State. The direct effect of the Regulation will mean its provisions will replace divergent data protection law in Member States. The Regulation will thus provide a new backdrop against which debates about data protection and biobanking will take place.

The Regulation applies in a broad range of contexts. Article 2(1) of the Commission’s versions outlines the scope of the proposed Regulation stating: ‘This Regulation applies to the processing of personal data wholly or partly by automated means, and to the processing other than by automated means of personal data which form part of a filing system or are intended to form part of a filing system’ (European Commission 2012a, Article 2(1)).^j^ The Regulation will apply to data processing conducted in relation to research and therefore in the biobanking context.

However, the Regulation does not apply when ‘personal data’ are not being processed. In this regard, there are two situations in which the scope of the concept of ‘personal data’ in relation to biobanking has been unclear – the case of anonymous data, and the case of the physical sample (as opposed to the extracted data). The authors would suggest that the scope of the Regulation should be given a broad interpretation. They believe that the concepts of anonymity (and pseudonymity) are not effectively applicable to genetic data or biobank processing. Accordingly the authors regard biobanks to always be process personal data and believe that the provisions of the Regulation should always apply.^k^ They also believe that the Regulation must apply to both physical samples, and associated data.^l^ In summary, the authors believe that the Regulation must apply to biobanking from the moment of collection of samples and data, and throughout the lifetime of storage and further processing of all samples and data.

When the Regulation is found to apply *rationae materiae*, it must then be defined how it applies. The Regulation operates a two tiered system of protection. This system classifies data according to how much of an impact processing could have on the fundamental rights of the data subject. The first tier is for ‘normal data’. The second tier is for ‘sensitive data’. A stricter set of conditions and a higher standard of oversight are applied to the processing of sensitive forms of data. A list of sensitive types of data is laid out in Article 9 of the Commission’s version (European Commission 2012a). The categories mentioned in Article 9 of the Commission’s version include; ‘personal data, revealing race or ethnic origin, political opinions, religion or beliefs, trade-union membership, and the processing of genetic data or data concerning health or sex life or criminal convictions or related security measures’.^m^ Article 4(10) of the Commission’s version defines genetic data: “genetic data’ means all data, of whatever type, concerning the characteristics of an individual which are inherited or acquired during early prenatal development’ (European Commission 2012a, Article 4(10)). Interestingly, the Parliament suggest an alteration to this definition: “genetic data’ means all ***personal*** data ***relating to*** the ***genetic*** characteristics of an individual which ***have been*** inherited or acquired ***as they result from an analysis of a biological sample from the individual in question, in particular by chromosomal, desoxyribonucleic acid (DNA) or ribonucleic acid (RNA) analysis or analysis of any other element enabling equivalent information to be obtained’***. In turn, the Council suggest that: “genetic data’ means all ***personal*** data ***relating to the genetic*** characteristics of an individual ***that have been inherited of acquired, resulting from an analysis of a biological sample from the individual in question***’. However, the Council extend this definition in their amendment to Recital 25a which states that genetic data can be obtained not only from analysis of a biological sample, but also; ‘in particular by chromosomal, deoxyribonucleic acid (DNA) or ribonucleic acid (RNA) analysis or analysis of any other element enabling equivalent information to be obtained’.

Under the Commission’s definition, every type of data collected by a biobank might be considered as ‘genetic’. We have already argued that the physical sample should be considered as data, and this would accordingly be considered data which ‘concerned’ inherited characteristics – the genome would be extracted from the raw sample. Any data which were collected through the analysis of the sample would also be data which ‘concerned’ inherited characteristics – the genome itself. Finally, the biobank collects other forms of data from the individual – for example health and lifestyle data. Many phenotypes result from the interaction between the individual’s genetic architecture and environmental factors. The biobank collects these other types of information to study this interaction – how non-genomic factors affect gene expression. Information collected with the intention of analysing gene expression, even if not necessarily ‘genetic’ on its own, would become information ‘concerned’ with genetics through the context of analysis. Under this definition, all data collected by the biobank, from the moment of collection, would be regarded as ‘genetic data’ and therefore must be treated as ‘sensitive data’.

Under the Parliament and Council definition however, it would only certainly be the results of the analysis of the biological sample which would certainly be defined as ‘genetic’. An argument could be made that, when other forms of data are subjected to an analysis which aimed to reveal information about genetics, they could be conceived of as ‘other elements’ allowing information about inherited characteristics to be obtained. In this reading, such data would also, following analysis, be regarded as ‘genetic data’. However under the Parliament and Council definitions, before such an analysis was conducted, no collected data would be regarded as ‘genetic’. Thus the physical sample would not be regarded as ‘genetic’, nor would any of the other information collected.^n^ Nevertheless, even under the Parliament and Council versions of the Regulation, such other information could be conceived of as ‘health data’. Both versions regard health data as any data which relates to the physical or mental health of an individual. As biobanks are part of the health research infrastructure and all data will be used in health research, this information can be regarded as ‘relating’ to health. Accordingly such data should also be regarded as ‘sensitive’.

As data collected and processed by biobanks is classified as ‘sensitive’ under Article 9, ts processing is generally prohibited under 9(1); ‘the processing of [sensitive data] shall be prohibited’, subject only to certain limited exceptions laid out in Article 9(2) (European Commission 2012a, Article 9(2)). Accordingly, for biobanks to legitimately process data, they need to do so under an applicable exception under Article 9(2).^o^

### Consent as the relevant exception for biobanks under article 9(2)

Article 9(2) offers a number of possible exceptions. Article 9(2)(a) states that the consent of the data subject allows the processing of sensitive data.^p^ Article 9(2)(b) – (j) list a number of other justifications. The exceptions under 9(2)(b)-(j) can be loosely categorised as ‘public interest’ exceptions. Public interest exceptions take the decision as to whether data should be processed away from the individual – the normative benefit of processing has already been defined in the political process (Tene & Wolf 2013, pp 3–4). There has been considerable debate as to which form of justification must be relied upon where more than one ground seems relevant. In the case of biobanking, the authors feel that there is a lexical order to legitimating conditions. In this regard, they feel that consent under 9(2)(a) is the relevant exception that ought to be relied upon.

In particular, there are two strong legal theoretical arguments suggesting the primacy of consent over other possible justifications.

First, there is an argument from human rights jurisprudence. One of the main goals – arguably the main goal – of the Regulation is to provide a framework to protect individuals’ rights. Sensitive data listed under Article 9 are those data whose processing constitutes a specific risk to rights. Commentators suggest that such data are, by *nature* capable of infringing on fundamental rights. Whenever they are processed, there is thus a *prima facie* infringement of the rights of the data subject (Beyleveld 2004a, pp 10–11). In the European Court of Human Right’s case law, a substantial distinction is thus made between the choices of justification when processing sensitive data. In *M.S. v. Sweden*, the Court found that the lack of consent of the data subject to the disclosure of certain sensitive data was a key element in the finding of an infringement of Article 8 (the right to privacy). Accordingly, the plaintiff’s consent would have served to waive the existence of an infringement (M.S. v. Sweden, European Court of Human Rights 1997, §34-35). Thus, if consent is relied upon under 9(2)(a), there is no interference with the data subject’s rights. If a public interest is relied upon under 9(2)(b)-(j) there is an interference, it simply has a justification which overrides the right. In human rights law, if the same result can be achieved with less impact on an individual’s rights, this option must be taken. Thus, in relation to the underlying rights at stake, if consent can be obtained – which it can in biobanking – this is the ground under 9(2) which should be relied upon (Beyleveld 2004a, pp 10–11).^q^

Second, in the ethical and legal tradition of biomedical research, the research subject’s consent – under normal circumstances – is a prerequisite to conducting research. Biobanking falls definitively within the sphere of medical research. Whilst there is argument as to the form of consent necessary, there is little dispute that biobanks collecting samples directly from research subjects should obtain consent. Theoretically, the use of consent in medical research is a reflection of the autonomy, and primacy of decision, of the research subject in the research process. This has a long history, emerging form hard lessons drawn from horrific instances of paternalism and abuse in research.^r^ Given that the desire of the potential research subject plays the central role in contemporary thinking about biomedical research, the use of a public interest exception under data protection law to allow research when the data has been collected from the individual seems awkward.^s^

It has been established that biobanks ought to rely on the consent of the data subject. However, the Regulation lays out a number of further conditions defining when a consent is legitimate under 9(2)(a). One sub-set of these conditions relates to what the data subject must know about the proposed processing before they can be seen to legitimately consent. Article 4(8) states ‘the data subject’s consent’ means any…specific [and] informed…indication of his or her wishes’ (European Commission 2012a, Article 4).^t^

Where there is an obligation to inform, there must be a set of criteria against which the discharge of this obligation can be measured. In this regard, Kosta states: ‘Concretising these questions in the area of data protection, it is essential to examine [1] what kind of information and [2] how much of it does the data subject need [− how specific does information need to be]’ (Kosta 2011, p 178). Using these questions as a baseline, it is possible to infer from the text of the Regulation, and from certain relevant jurisprudence, a set criteria which constitute the nuts and bolts of this obligation.

### Which categories of information must be provided to the data subject?

In relation to Kosta’s first category, the Articles in the Regulation directly elaborating the conditions of consent do not clarify which kinds of information must be provided to the data subject.

However, elsewhere in the Regulation, general informational obligations (relevant to all processing situations) are elaborated. These obligations must also be met in processing legitimated by the data subject’s consent (Beyleveld 2004b, pp 69–71). The most important Article in this regard is Article 14. Article 14(1) elaborates a number of categories of information which must be given, by the data controller, to the data subject, when data is collected directly from the data subject – as in the case of open consent:^u^

- 14(1)(a): ‘the identity and the contact details of the controller and, if any, of the controller’s representative and of the data protection officer’.
- 14(1)(b): ‘the purposes of the processing for which the personal data are intended’.
- 14(1)(c): ‘the period for which the personal data will be stored’.
- 14(1)(d): ‘the existence of the right to request from the controller access to and rectification or erasure of the personal data concerning the data subject or to object to the processing of such personal data’.
- 14(1)(e): ‘the right to lodge a complaint to the supervisory authority and the contact details of the supervisory authority’.
- 14(1)(f): ‘the recipients or categories of recipients of the personal data’.
- 14(1)(g): ‘where applicable, that the controller intends to transfer to a third country or international organisation and on the level of protection afforded by that third country or international organisation by reference to an adequacy decision by the Commission’. Importantly, the Parliament’s version adds to that ‘***in case of transfers referred to in Article 42, Article 43, or point (h) of Article 44(1), reference to the appropriate safeguards and the means to obtain a copy of them***’ must also be provided. The Council version removes the necessity to provide any information as to the protection standard in third countries.
- 14(1)(h): ‘any further information necessary to guarantee fair processing in respect of the data subject, having regard to the specific circumstances in which the personal data are collected’ (European Commission 2012a, Article 14).

The Parliament’s Consolidated version adds further categories of information to this list:

- 14(1)(ga): ‘where applicable, information about the existence of profiling, of measures based on profiling, and the envisaged effects of profiling on the data subject’.
- 14(1)(gb): ‘meaningful information about the logic involved in any automated Processing’.
- 14(1)(ha): ‘where applicable, information whether personal data was provided to public authorities during the last consecutive 12-month period’.

The Council further add:

- 14(1a)(b): ‘where the processing is based on point (f) of Article 6(1) [on the grounds of the legitimate interest of the controller], the legitimate interests pursued by the controller’.^v^

Consent as a legitimate ground to process sensitive data is not new in the Regulation. The same possibility existed in the Directive under Article 8(2)(a). Accordingly, it is legitimate to consider European level interpretation on conditions of consent in the Directive as having continued relevance in understanding similar conditions in the Regulation. Of particular importance is the guidance on consent provided by the Article 29 Data Protection Working Party (2011). They suggest further categories of information which must be provided. In particular, they state: ‘[consent] includes notably which data are being processed’ (Article 29 Data Protection Working Party 2011, p 17).^w^ The Article 29 Working Party also state that consent ‘should refer clearly and precisely to the scope and consequences of processing’ (Article 29 Data Protection Working Party 2011, p 17). The clarity of scope of processing is not a category of information itself. It is a description for the composite of information in all other relevant categories. This is also, to a certain extent, true for the category of ‘consequences’, although it is also possible to consider ‘consequences’ as a separate category in its own right. Known risks or benefits ought to be communicated to the data subject.^x^

### How specific must information provided to the data subject be?

Defining rules, even general rules, for Kosta’s second question – how much information does the data subject need, or how specific should it be – is much more difficult. The Regulation itself is quiet on this point. The core problem in this regard is that specificity of information can only really be measured against a type of information in a given context. However, context cannot be the only relevant factor in the consideration of specificity of information. This would render the provisions of the Regulation meaningless.

In this regard, the Article 29 Working Party have attempted to provide guidance which may be relevant across contexts.^y^ They suggest that the specificity of information should depend on how *complex* processing is. ‘The more complex data processing is, the more can be expected from the data controller. The more difficult it becomes for an average citizen to oversee and understand all the elements of the data processing, the larger the efforts should become for the data controller to demonstrate that information was provided based on specific…information’ (Article 29 Data Protection Working Party 2011, p 20).

The Article 29 Working Party does not mention the potential rights impact of processing as relevant to the specificity of information. However, the aim of consent is to provide legitimacy for an action which would otherwise infringe an individual’s rights. Accordingly, Dammaan and Simitis interpret the level of specificity of information required as also being dependant on the *potential impact on the rights of the data subject* (Dammann and Simitis 1997, p 115).

Defining the complexity of biobank processing is difficult. There is little jurisprudential indication as to what aspects of processing need to be taken into account when evaluating complexity. However, the authors feel that there is a strong argument to suggest that biobanking processing should be regarded as comparatively complex. First, the processing is done on a unique form of data – genetic data. Second, the framework used to interpret this data is equally unique – comprising of a complex and esoteric science. In this regard, studies have shown the comprehension of the public of the specificity of genetic data, and the processing of genetic data, as quite limited (Lanie et al. 2004). Third, in an open consent model, the networks through which data will travel are multifaceted. In turn, there is no given limit as to how often a genetic sample/data can be used (although limits may be implicit in the physical quantity of sample collected and in how it is used) nor to how many researchers can apply to use it. Fourth, each of these researchers may be processing the data with different goals in mind and thus process the genetic data to reveal different types of information about the data subject. Accordingly, there is no set limit on how much, or which type of information, might be eventually extracted from initially collected material. Finally, there are only a very limited number of situations in which an individual might have their genetic data processed, and almost no situations in which the individual might have so much genetic data processed. This renders the processing taking place in the biobanking context highly unusual. Accordingly, it is unlikely that many people will have much experience, or a solid frame of reference, through which to interpret and evaluate such processing.

Consideration of the potential impact on the data subject’s rights through processing is a little easier. Here, there are normative reference points which clarify that the processing of genetic data represents a high risk to the rights of the individual. The Regulation – in Article 9 – provides normative clarification of the sensitivity of genetic data, whilst other authoritative legal sources go one step further and suggest that genetic data is data of ‘unique’ sensitivity. For example, the Article 29 consider genetic data as exhibiting characteristics which make it particularly sensitive (Article 29 Data Protection Working Party 2004a, pp 4–5). Equally, In the *Marper* case, for example, the ECtHR specifically recognized genetic data as ‘intrinsically private’ and gave a number of reasons for genetic data’s specific sensitivity. (S. and Marper v United Kingdom, European Court of Human Rights 2008, § 104).

The above only provides rough guidance. It does not describe the precise level of specificity of the information which must be given by the biobank. However, such guidelines do provide indicators which can be considered comparatively. If it is accepted that biobank processing is both complex, and involves the processing of data of a highly private quality, it is logical to consider that the level of specificity of information required from the biobank ought to be comparatively high in relation to other processing situations. Accordingly, jurisprudence relevant to processing situations with similar, or lower, specificity thresholds can be used to provide relevant points of comparison.

With regard to these framework conditions, the authors see problems for biobanks to provide the relevant specificity of information under an open consent procedure in relation to at least four categories of information; purpose, recipients, third country transfers, type of data and consequences of processing.^z^

### Purpose

Under Article 14(1)(b)^aa^, the biobank has the obligation to provide the data subject with information as to ‘the purposes of the processing for which the data are intended’ (European Commission 2012a, Article 14(1)(b)).

It is clear that blanket consent is prohibited (requesting consent to all future processing purposes). ‘Open consent’, however, is clearly not ‘blanket consent’. The scope of open consent is not to seek justification for every possible future use of data, but to seek justification for the future use of personal data *in research*. The UK Biobank, in this regard states: ‘Information and samples from UK Biobank participants will be available only to researchers’ (UK Biobank 2010).

However, simply as a biobank can elaborate a broad purpose of ‘research’, and does not seek ‘blanket consent’ does not mean it has adequately fulfilled its obligations under 14(1)(b). ‘Research’ is not a monolithic description of purpose. In fact, it can be better described as ‘sectoral’ description of purpose (Nomper 2005, pp 172–174). Chapter IX of the Regulation elaborates specific rules for certain forms of data processing. Amongst these situations in the Commission’s version, for example, are health (Article 81), employment (Article 82) and research (Article 83) (European Commission 2012a, Articles 81, 82, 83). ‘Research’ can be further subdivided, along a number of axes, into many sub-categories – for example, aids research, cancer research, stem cell research. Each of these categories could be further sub-divided, and so on.

Unfortunately for biobanks, available guidance related to the specificity of purpose in processing suggests that information only outlining a ‘sectoral’ purpose is insufficient. First, such guidance is available in relation to the concept of ‘purpose limitation’. The concept of ‘purpose limitation’ operates to set limiting conditions on the scope of processing. It is one of the general principles of data processing laid down in Article 5 of the Regulation and is applicable to all processing situations.^bb^ Although it does not directly clarify the conditions of consent, the concept of ‘purpose limitation’ in Article 5 and the concept of ‘purpose specificity’ in relation to 9(2)(a) are tightly linked (Article 29 Data Protection Working Party 2013, pp 13–14). Guidance related to one can act as a guideline as to how to interpret the other. In this regard, the Article 29 Working Party have stated that ‘[v]ague or general purposes such as ‘improving users’ experience’, ‘marketing’, ‘IT-security’ or *‘future research’* will – without more detail – usually not meet the criteria of being ‘specific’ [emphasis added] (Schulte in den Bäumen et al. 2010, p 52). Second, there is relevant guidance directly related to the conditions of consent. Of particular relevance in the case of biobanking, is the exemplary guidance on consent and Electronic Health Records (Article 29 Data Protection Working Party 2007b). This is particularly relevant as it refers to a situation in which data classified as ‘sensitive’ by the Regulation are processed. The Article 29 Working Party state that: ‘consent must relate to a well-defined, concrete situation in which the processing of medical data is envisaged (Article 29 Data Protection Working Party 2007b, p 9). Therefore a ‘general agreement’ of the data subject e.g. to the collection of his medical data for an EHR and to subsequent transfers of these medical data of the past and of the future to health professionals involved in treatment would not constitute consent’.

### Future recipients of data

Article 14(f)^cc^ requires the biobank to provide the subject with information as to ‘the recipients or categories of recipients of personal data’. (European Commission 2012a, Article 14(f)).

It is clear that the biobank cannot list, at the moment of consent, the precise set of future recipients. Only after open consent has been given will researchers, and research projects, begin to apply to use the material. However, the biobank *can* offer information as to ‘the categories of recipients’. As the purpose is ‘research’, the recipients of samples can be described under the category of ‘researchers’. The UK biobank does precisely this when stating that ‘Information and samples from UK Biobank participants will be available only to researchers’ (UK Biobank 2010, p 8).^dd^

The category ‘researchers’ is, however, very broad. The category of ‘researchers’ could consist of a number of subdivisions – researchers at institution X, researchers in project Y cancer researchers, stem cell researchers etc. Once again, each of these categories could be subdivided, and so on. The question is thus the same as the question that arose in relation to purpose. Can we say that the category ‘researchers’ is sufficiently specific?

The Article 29 Working party opinion on Electronic Health Records provides an indication that it is not. The opinion states: ‘a ‘general agreement’ of the data subject e.g. to the collection of his medical data for an EHR and to subsequent transfers of these medical data…to health professionals…would not constitute consent’ (Article 29 Data Protection Working Party 2007b, p 9). In this example, there is reference to a broad category of recipients – ‘health professionals’ – as part of reason for the failure of consent. From the example it is not conclusive that consent would fail solely due to the breadth of class of recipient. However, in combination with a lack of specificity of purpose, the consent certainly fails.

In another example, the Article 29 Working Party considers the example of passing personal data to third parties for the purposes of direct marketing. They suggest that if user consent is obtained, then data – for example e-mail addresses – may be given to undefined third parties in the future (Article 29 Data Protection Working Party 2007b). This has been used as an argument to suggest that the data subject could consent to the passing of their data to a broad category of undefined future recipients. This would open the door to the biobanks to rely on ‘researchers’ as a category of sufficient precision (Nomper 2005, pp 172–174). However, this analysis is only partially correct. In their Opinion, the Working Party go on to state that, even if the future recipients of data are not specified, ‘the information provided to the data subject should then indicate the purpose(s), the goods and services…for which those parties would send e-mails’ (Article 29 Data Protection Working Party 2004b, p 5). Accordingly, a consent suggesting the passing of personal data to a group of future, undefined, recipients, could only be regarded as legitimate if the purpose for which these recipients would be allowed to process data were specific. As above, this is not possible for the biobank to do.

### Third country transfers

Article 14(1)(g)^ee^ of the Commission’s version requires the biobank to provide the data subject with information as to ‘the plans of the controller to transfer data to third countries, which countries and the details as to the adequacy of these countries’ data protection arrangements’ (European Commission 2012a, Article 14(1)(g)). The Parliament’s version adds that reference to other mechanisms allowing cross-border data transfer would also suffice. Interestingly, the Council’s version removes the obligation to provide information on the adequacy of standards of protection in the third country.

Of course, provision of information in this category is not *necessarily* problematic for an open consent procedure. The requirement to provide information in this category only becomes relevant when transfer of samples or data is envisaged to third countries outside the EU. This means that any EU biobank operating open consent procedures, planning only to provide samples and data to domestic or European researchers, and to prohibit these researchers from further circulating this data to third countries outside the EU, need not worry about this requirement.

However, large scale biobanks, and the research communities they serve, are becoming more international. It is questionable whether such biobanks will operate domestically in reality. The larger the biobank becomes, the more likely this is to be the case. The Geenivaramu supports projects from around the world as does the UK Biobank. The UK biobank explicitly states ‘bona fide researchers working in countries around the world will be able to apply to use the resource’ http://www.ukbiobank.ac.uk/all-faqs/ (last consulted 27.11.2013). In its consent materials the data subject is simply informed, in a broad fashion, that data may be transferred to third countries in the context of future use (UK Biobank 2010, p 8).

Open consent procedures are in place precisely as it is not desirable, at the time of collection, to state recipients of data. If it is not possible to list applicants or recipients of data in advance, then it is equally impossible to list the countries of origin of those recipients. This is problematic under every version of the Regulation.

Needless to say, if the countries cannot be named, then the level of protection (or any relevant adequacy agreements) cannot be named either. This is difficult to reconcile with Article 14(1)(g) of the Commission and Parliament texts. The Council version would seem to be a little more conducive here as it does not mention information as to adequate protection. Nevertheless, jurisprudence on cross-border data transfers from the Article 29 Working Party – which would still be applicable even in the case the Council’s wording was adopted – states; ‘the data controller must be able to prove in all cases that, firstly, he has obtained the consent of each data subject and, secondly, that this consent was given on the basis of sufficiently precise information, including information on the lack of protection in the third country’ (Article 29 Data Protection Working Party 2005, p 5).

### Data collected

At the moment of collection, the biobank must give the subject a specific description of the data to be processed. Some data processed by the biobank may be collected through application forms or in discussion with participants.^ff^ This data can be regarded as ‘normal data’ – Manson refers to this as ‘communicative data’ (see section 13). It is in the form of comprehensible information and communicates limited and defined knowledge about the data subject. The biobank can be specific with regard to this data. However, the main source of biobanks’ value is in the biological samples they collect and the genetic data contained therein. The whole genome is contained in this sample. Accordingly, at the moment of collection, the biobank could be no more precise than to state: a source of genetic data, containing the whole genome, has been collected. This raises at least two questions.

First, the possession of an individual’s genome is the possession of a source of further data about the individual. A genome is extracted from DNA. DNA has four different chemical building blocks – bases. The average human genome has approximately 3 billion base pairs. The term ‘genetic data’ could refer to any number of bases, up to the complete genome. Whilst the sequenced bases themselves constitute personal data, a series of letters is largely irrelevant (unless the series can be used as a unique identifier).^gg^ However, when the relevant interpretative frame is applied to a given set of bases, a number of individual characteristics which are seen to have a genetic basis can be analysed. There are a huge number of traits which can potentially be analysed from any given sample. These vary broadly in form, describing not only physical attributes (blue eyes), but also current, or future, medical (has condition A; does not have condition A, is P% likely to contract condition A in the future) or social status (has genetic predisposition toward social characteristic S) (Taylor 2012, pp 41–53).

The only factor serving to group these traits is their source material. Accordingly, whilst ‘genetic data’ is listed as a category of data in the Regulation, the authors do not regard this as a useful, or specific, description of data which has been collected. By claiming that ‘genetic data’ had been collected, a biobank would be doing nothing more than referencing source material. This seems very difficult to reconcile with the obligation to give specific descriptions of data which had been collected and were to be processed.

Second, the content and amount of information capable of being extracted from the genome at any given time, is dependent on the state of genetic science at that time. Accordingly, developments in genetic science will expand the content and form of information about the individual which can be extracted from the sample.

It could be argued that future uncertainty is inherent in all transactions, data based or otherwise, consent based or otherwise. However the significance of the future potential of genetic data has received specific legal recognition. The ECtHR observes this feature of genetic data as integral to its sensitivity and its interaction with individual rights. The court states: ‘Indeed, bearing in mind the rapid pace of developments in the field of genetics and information technology, the Court cannot discount the possibility that in the future the private-life interests bound up with genetic information may be adversely affected in novel ways or in a manner which cannot be anticipated with precision today’ (S. and Marper v United Kingdom, European Court of Human Rights 2008, § 71). Accordingly, this is a feature of genetic data which demands serious consideration in any processing context.

Open consent would allow the continued processing of data into the future, regardless of changes in genetic science. The UK Biobank state: ‘during follow-up over the next few decades, your stored samples will be analysed for approved health-related research’ (UK Biobank 2010, p 3). For consent to provide legitimation to process data, this consent must be based on accurate information. For consent to continue to provide legitimation over time, the information on which it was based must remain accurate. Accordingly, there will be some point at which the science behind processing has so materially changed data which can be extracted from the sample, that the initial consent cannot be held to be valid – if it ever was.^hh^

The above analysis paints a negative picture for open consent. Under the conditions laid out in the Regulation, open consent looks unlikely to succeed.

However, this is not the end of the road for open consent. Before it can be concluded that open consent is incompatible with data protection law, a further set of arguments must be considered. Proponents of the open consent process have suggested that there are mechanisms in data protection law which may serve to relax the problematic informational obligations outlined in sections 6 to 9. This set of arguments can be related to a third question Kosta asks: ‘to what extent [do] the informational rights of the data subject or obligations of the data controller…influence the information that should be provided’ (Kosta 2011, p 176).

Two arguments in particular have been put forward. First, that the expectations of the data subject can serve to relax informational obligations. Second, that the other protections provided by biobanks can serve as a substitute for the provision of specific information.

### Do the expectations of the data subject serve to relax conditions of consent?

This first argument suggests that the data subject has the right to alter the relevant rules on consent. Thus, should the data subject have a certain reasonable expectation of processing which will occur, the data subject has the right to enter into this transaction despite the legal limitations which would otherwise prohibit such a transaction. So, the data subject could allow open consent, even though it may violate the conditions laid down by the Regulation.

Although the idea that the data subject could alter the requisite informational obligations finds no expression in the Regulation itself, it does finds some justification in data protection jurisprudence. The Article 29 Working Party clearly states that the ‘granularity’ – a word often used when referring to specificity – of a given consent should be ‘based on the reasonable expectations of the parties’ (Article 29 Data Protection Working Party 2011, p 17). However, the extent to which conditions can be changed on the grounds of ‘reasonable expectations’ is unsure. The Article 29 working party foresees the expectations of the parties as only one factor relevant in determining the informational obligations of consent – the other half being the objective criteria laid out in sections 4 and 5, above.

In the opinion of the authors, it seems unlikely that the expectations of the parties was ever meant to function as the *sole*, or *main*, principle in deciding the relevance of statutory informational obligations in a given situation. This would have the effect of rendering the obligations and conditions laid out in the Regulation, and elsewhere, meaningless. Allowing open consent on the basis of the desire of the data subject would go far beyond taking the data subject’s expectations *into account.* It would constitute granting the data subject the right to remove statutory conditions on consent.

### Do safeguards serve to relax conditions of consent?

As well as obtaining consent to process data, biobanks may employ a number of other ‘buffer safeguards’ which aim at avoiding privacy harms to the individual. These include technical measures, such as data security systems, pseudonymisation and anonymisation of data. They also include organizational approaches. For example, governance systems – including ethics committees – which take data subject’s rights into account when deciding on biobank activity. The argument follows that the presence of such ‘buffer safeguards’ should allow the relaxation of the informational conditions on the initial consent.

Generally speaking, the reduction of risk through technical or organizational measures is well recognized in data protection law. Indeed, risk reduction measures can be relied on as grounds to reduce other obligations in data protection law. For example, anonymisation of data voids the applicability of data protection law. Indeed, the Article 29 Working Party suggests, in relation to pseudonymized data, that ‘the risks at stake for the individuals with regard to the processing of such indirectly identifiable information will most often be low, so that the application of [data protection] rules will justifiably be more flexible than if information on directly identifiable individuals were processed (Article 29 Data Protection Working Party 2007a, p 18).

However, how risk reduction interacts with the requirements for legitimate consent in the Regulation is uncertain. There is clearly some connection. For example, sensitive data represent a greater risk, and therefore it more specific information should be provided be the data controller. Nevertheless, there is little in the current law to support the argument that buffer safeguards should serve to reduce relevant consent requirements.

Formally, there is no reference in the Articles referring to the conditions of consent, nor in Article 14, to a reduced standard of obligation based on risk reduction mechanisms being in place.^ii^ In turn, the authors know of no relevant jurisprudence defining how, or how far, such risk reduction approaches can function to change the relevant conditions of consent. Finally, certain risk reduction mechanisms are statutorily obligated in all processing contexts anyway. For example, in Article 30, the Regulation lays out the conditions on privacy by design. The controller is obligated to institute ‘organizational and technical measures’…‘proportionate to the risks [of processing]’ (European Commission 2012a, Article 30). It is difficult to see why, without specific mention, that the presence of such mechanisms should ever allow a relaxation of other statutory obligations – for example those on consent.

Even if one accepts arguments that risk reduction approaches could reduce the relevant standard of information required, it is not clear that they would always be applicable in the biobanking context. There is doubt as to how effective certain risk reduction approaches can be in the biobanking context. In section 2, we have already discussed our skepticism at the idea of anonymisation and pseudonymisation in genetic data and in biobanking. Lunshof et al. generally suggest that claims as to the effectiveness technical approaches to risk reduction procedures in large scale biobanks may be greatly exaggerated (Lunshof et al. 2008, pp 4–6). Nor are the authors convinced that governance systems, such as ethics committees, can be truly regarded as risk reduction mechanisms. Governance systems are essentially oversight and proportionality mechanisms. In this regard such mechanisms take decisions as to whether processing is justified without the individual’s involvement. Proportionality mechanisms, may, but do not necessarily, reduce overall risk for the individual (Petrini 2010, pp 217–220).

### Lex ferenda – a change of consent conditions for biobanks?

The analysis above concludes that open consent cannot meet the standards demanded by the Regulation.

However, the problematic informational obligations are not rights, which *prima facie* need to be protected. They emerged from the legislative process. They are results of political consideration as to the function of the consent, and the need to balance rights and interests involved in each consent transaction.

The authors would argue that there are a number of features of the biobanking context, and of genetic data, which do not fit the assumptions made in the legislative process. Accordingly, the reasoning behind the approach to, and strictness of, the conditions laid out in the Regulation, may not be relevant to biobanking. In turn, there is ground for the political reconsideration of the conditions relevant to consent in biobanking.

Two arguments are put forward: First, that the specificity threshold has been set in relation to a different context, and set of rights and interests, than those prevalent in biobanking. Second, that the approach taken to consent in the Regulation is poorly applicable to any instance in which a genetic sample, or genetic data, is processed.

*De lege ferenda,* such argumentation supports a call for a debate on biobank specific consent conditions. The elaboration of such sector specific rules is not only feasible, it is encouraged under the Regulation. Indeed, one of the main driving factors for data protection reform was that the Directive lacked the flexibility to deal with novel processing situations.

In terms of legal procedure, there are a number of ways such a set of rules could be brought into existence. The Regulation is still before the Council and changes can still be made as part of the normal legislative process. Equally, the Regulation conceives of a number of mechanisms for interpretation and adaptation to specific, or novel, processing scenarios – for example the consistency mechanism laid out in Chapter VII and the establishment of the European Data Protection Board. Finally, in the original version of the Regulation, the Commission was given the power, under Article 9(3) to; ‘further [specify] the criteria, conditions and appropriate safeguards for the processing of the special categories of personal data referred to in paragraph 1 and the exemptions laid down in paragraph 2 [of Article 9]’ (European Commission 2012a). The European Data Protection Board is granted a similar power in the Parliament’s Consolidated Version.

### Where does the regulation’s concept of ‘specific and informed’ come from: does this remain relevant to biobanking?

In relation to the underlying rights data protection law seeks to protect, the original position is that the data subject has the right to informational self-determination. Without justification, this right should not be limited. In essence, the data subject has ‘the right to take a risk’ and to do whatever they want with their data regardless of uncertainty or potential future risk (Schulte in den Bäumen et al. 2010, pp 39–40; Taupitz & Weigel 2012, pp 265–266). This is exemplified by Article 9(2)(e) which allows the possibility for the data subject to ‘manifestly’ make their data public. The data subject thus has the ability to publicly release their genetic code and all information which the biobank would collect in an open consent procedure. A far more intransparent and risky act.

Consent, however, is a transaction between two parties which allows one party to engage in an action which would otherwise be prohibited. In this regard, it is the manifestation of the right to informational self-determination in the Regulation. A central condition for all consent transactions is that the parties transact on the basis of relevant knowledge and understanding (Beyleveld & Brownsword 2007, pp 145–146). This is the rationale behind the obligation to obtain ‘specific and informed’ consent.

There are two general reasons legislators choose to specify more precise statutory conditions relating to informational criteria in consent transactions. First, these may be necessary to provide a standard framework in which a transaction can take place – a set of rules for a legitimate interaction. Second, they may be necessary to correct for imbalance in the context in which consent takes place. In particular, they may be needed to correct for the danger that that the consent mechanism allows one party to unfairly profit at the expense of the other. In the context of the Regulation, the informational obligations related to ‘specific and informed’ consent are tightly tied to this second justification. In particular, the data subject is seen as a vulnerable party in need to protection.

However, the setting of legislative standards on relevant knowledge and information, must also be considered in the light of the trade off it entails. On the one hand, the more information that must be provided to the data subject, the more ‘informed’ that data subject might be. On the other hand, the harder it will be for any party to fulfil the requirements of a legitimate consent, and the more limited the scope of actions permissible under consent will be – eventually limiting the data subject’s choice, and right to informational self-determination, as well.

In the legislative discussions leading to the drafting of the Regulation, there was a focus on consent, and on the conditions of consent (European Commission 2012b). Such discussions predominantly revolved around the online context – particularly social networks. Of course, this is not the only area covered by the Regulation. However, its prevalence in the legislative discussion is indicative of the perception of the relevant context and interests. Certain important features of the discussion of the online environment stand out as significant. First, the interests of the parties involved in typical data processing activities online – for example social networking or e-commerce – can be expressed in adversarial terms. As personal data form the basis for the business models of many companies, the right to privacy of the data subject may be in direct opposition to the economic interest of the data controller. Second, when personal data are processed in an online environment, they often have value in so far as they can be used, whether directly or indirectly, to leverage benefit against the data subject (or a group of data subjects) (Manson & O’Neill 2008, pp 115–119). Finally, there was much discussion as to the intransparency of the online environment, and how this made it difficult for the data subject to understand what they were consenting to.

In relation to the above, certain conclusions can be drawn as to the concept of uncertainty in consent in the mind of the legislator. First, a lack of information – uncertainty – is to the disadvantage of the data subject as it provides the space for data controller to leverage advantage. Second, uncertainty on the part of the data subject may not imply uncertainty on the part of the data controller. In this case, uncertainty is not neutral, but obscures a power/information imbalance – facebook may have all the relevant information about the context in which it will process, it is unlikely that the data subject has this same information. In turn, this has a substantive bearing on the ability of the data subject to understand the processing and to understand its consequences. Accordingly, the desire of the legislator to protect the data subject uncertainty is understandable. The trade-off in this case shifts toward stricter informational requirements as a response to the dangers of uncertainty, albeit at the expense of informational self-determination.

The biobanking context is rather different. First, the two interests which are directly at stake in biobanking are the privacy of the individual, and the public interest in medical research. There is not the same type of adversarial relationship which exists between these interests as exists in the online context.^jj^ Indeed, given that participants’ trust in researchers, and scientific research in general, is central to effective research, one might even talk of a confluence of interests. Laurie states: ‘this…does not seek to set interest against interest but seeks to bring about a confluence of public and private interests. In this sense it is a unique construct’ (Laurie 2002, pp 165–167). Second, data processing done in biobanking is not aimed at any individual, nor is it done with any intention to have any effect on an individual. In fact, the individual is only relevant as a point of administrative reference – character, identity and personality are irrelevant. The product is impersonal and undirected scientific knowledge (Manson & O’Neill 2008, pp 115–119).

In an open consent procedure, uncertainty might thus be viewed rather differently. First, the lack of information is not necessarily to the disadvantage of the data subject, as it is unlikely to be used to leverage advantage against the data subject. Second – provided that the transaction is governed by truth and transparency – the lack of information does not serve to obscure a relevant power/information imbalance. In turn, the lack of information does not prevent the data subject from making a choice, having been given all current, available and relevant information about the proposed processing. In this reading, there is far less reason to protect the data subject through restrictive informational obligations. Accordingly, one might argue that the trade-off should slide the other way – toward looser informational obligations and a more permissive approach to allowing the data subject to decide what happens to their data.

However, this is not the end of the debate. Whilst it is true that the conflict of interests is qualitatively different in the biobanking context and the online context, this does not automatically mean open consent is legitimate.

First, there are other regulatory spheres in which open consent has received much attention; in particular, in relation to the law (and ethics) on biomedical research. There is still no consensus in this area about the legitimacy of open consent.

Second, there are a series of considerations which are specific to genetics and genomics which extend beyond the particular relationships involved in the consent transaction. For example, genetic data are inherited and are therefore may be held in common with others – for example blood relatives. This means that the processing of other individuals’ genetic data is implied by the processing of one research subject’s genetic data. Further, genomics research raises a number of social and ethical themes – eugenics, for example, looms large. A range of arguments based on consideration of harms to other parties could be put forward as an alternative justification for limiting the scope of consent; to protect against ‘uncertainty’ and ‘possible future harm’ as such.

Finally, the above argument has only functionality in relation to some categories of information. The requirement to provide specific information on international data transfers does not rest on argumentation as to the conflict or confluence of interests between data subject and data controller. Further, even if it was concluded that the current informational obligations ought to be relaxed, the problem of the collection of genetic data would remain. Even if conditions were to be relaxed, the biobank would still be unable to give a description of the data collected.

### The focus of consent, communicative data and genetic data

Manson argues that data protection law has been designed to deal with ‘communicative information’ (2009, pp18-21). The Regulation follows in this tradition. Communicative information is information which has already been arranged into a specific linguistic form, which allows transfer of certain knowledge. The knowledge in question in any data processing operation is thus perceived to be, to a large extent, a limitable and definable substance.

When designing rules regarding the scope of consent, the legislator must seek to approach the subject of consent in terms of definable, quantifiable features. To an extent, communicative data can play a role as such a feature. It is possible for a data controller to explain to the data subject the nature of the data which will be processed, and the knowledge it contains. The individual will in turn be able to conceive of the personal and social importance of the information and what processing of such knowledge might mean. Accordingly, the legislator focussed on an ‘act of processing data’ as the subject of consent, and relied on a specific description of the ‘personal data’ being processed as a definable, quantifiable feature of this ‘act’.

Biological samples, or raw genetic data, as collected by biobanks, cannot be classified as ‘communicative information’. Raw genetic material is not information which has been arranged into knowledge. It has only value for the communicative data it may reveal about the data subject in the future. A number of interpretative processes are still required to produce this. Despite its lack of imminent relevance, it potentially contains a large quantity of communicative data – a quantity which grows with scientific progress (section 9) (Article 29 Data Protection Working Party 2004a, pp 4–5).

Thus, raw genetic material is not a definable substance in terms of knowledge, and is not a substance which has a clear social or personal meaning. Accordingly, it is not a definable, quantifiable substance around which a scope of consent can be built. A data subject cannot understand the personal or social significance of an ‘act of processing’ if they do not know which data will be processed. In the same way a consumer might have difficulty in deciding on a purchase if they do not know how much the item costs.

Whilst the above observation does not necessarily support or reject open consent, it opens the door to consideration of alternative approaches to conceiving consent in relation to the processing of genetic data. Certain such approaches may not prove so obstructive to open consent.

Consenting to a specific ‘act’ is only one approach to the subject of consent. Beyleveld and Brownsword observe that ‘the paradigm of private empowerment involves an agent freely and with relevant knowledge consenting to the creation of a new relationship. The change of relationship might involve something as simple as a gift or a promise or the consent might signal a willingness to be bound by the rules of a game or the outcome of a voting process (Beyleveld & Brownsword 2007, pp 145–146).’ In the biobanking context, it has been suggested that, rather than focus on a specific ‘act’, the consent should be to a set of governance and decision making systems – the research/data subject submits to the ‘rules of the game’ (Nomper 2005, pp 83). As far as the authors are aware, there has been little consideration of the compatibility of data protection law with the possibility to provide ‘procedural consent’. Certainly, this seems an unlikely candidate for general application. On the other hand, there is no reason to believe that a procedural approach to consent – limited to the biobanking context and with the correct amount of careful deliberation and transparency – would necessarily be prejudicial to the data subject. Eventually, the focus on ‘data’ and the ‘act of processing’ are simply useful tools for approaching the interaction between consent, data processing and underlying rights. If the focus were to move from the act of processing to the decision making mechanisms defining whether processing occurs, there may be less obstruction to open consent, as the same decision making mechanisms would remain the same regardless of the proposed use, and therefore the consent would remain valid across use purposes.

## Conclusion

Biobanking opens up a number of possibilities in research. Unfortunately, such possibilities cannot fully be realised under the strict conditions on consent laid down by traditional research ethics. Accordingly, the concept of open consent was developed.

In an open consent procedure, the data subject gives their consent to the biobank collecting and storing their data and genetic material. In the same consent, the data subject gives their permission for stored data and materials to be used in future research. The specifics of the research and who will conduct it are not specified at the moment of collection.

As biobanks process personal data, it is necessary that their operation is legitimate under data protection law. European data protection law is currently undergoing a process of reform, the first results of which have been outlined in the Proposed Data Protection Regulation. This paper has considered what the prospects for open consent under the Regulation are.

First, it considered how open consent should be understood under the Regulation. In this regard, the authors have argued that the Regulation only provides the opportunity for biobanks to process data provided they have the consent of the data subject. However, this consent can only be regarded as legitimate under certain conditions.

Second, the paper considered one sub-set of these conditions which are regarded as particularly problematic for open consent. The Regulation provides that the data subject’s consent must be ‘specific and informed’. These obligations are comprised of criteria along two axes. The biobank is obliged to provide the data controller with certain categories of information. Information in each category must reach a certain level of specificity. The authors conclude that the biobank will have difficulty in being adequately specific in relation to at least four categories of information:

1. Given that data and samples are collected prospectively, there is no clarity as to which research projects they will feature in. Accordingly, the biobank cannot be adequately specific as to the purpose of processing.
2. As the biobank cannot be specific in relation to the purpose of processing, the biobank will not be able to be adequately specific as to the potential recipients of data.
3. If the biobank wishes to operate internationally, it cannot, in advance, list the countries to which data will be transferred.
4. Biobanks collect human samples – these contain the complete genomes of data subjects. There is a huge amount of data contained in the genome and the development of genetic science will allow ever increasing amounts of data to be extracted. Therefore the biobank cannot provide information as to which data have actually been collected.

Third, the article considered whether there were any mechanisms under data protection law which would allow a relaxation of these conditions for biobanks. Two arguments were considered: First, that the expectation of the data subject could serve to relax the relevant informational obligations; second, that the technical and organisational privacy protection mechanisms provided by the biobank should serve to relax the relevant informational obligations. The authors found certain support for these arguments in data protection law. However, the extent to which they would allow a relaxation of informational obligations seems limited. Accordingly, it seems unlikely they could be used to justify open consent under the Regulation.

Finally, given that open consent cannot succeed under the Regulation *de lege lata*, the authors considered whether there was any justification for a legislative reconsideration of the problematic conditions of consent in relation to biobanking. Two arguments support this. First, they suggest that the informational obligations of consent were designed to protect the data subject from risks inherent in the use of the consent mechanism in a specific context – exemplified by the online context. They argue that the biobank context is not comparable to this context and does not present the same risk to the data subject. Second, they suggest that the legislator drafted the Regulation based on certain assumptions as to the nature of ‘data’. The authors argue that these assumptions are difficult to apply to genetic data and accordingly, any consent which aims at defining an ‘act of processing’ will inevitably be unclear.

### Endnotes

^a^Legal analysis has been conducted into whether the current Directive allows for open consent. Such analyses have generally concluded that open consent policies cannot meet the requirements of consent under the Directive due to the lack of specificity of information communicated (Schulte in den Bäumen et al. 2010). Certain of these analyses consider that open consent could be legitimated under public interest exceptions – for example that laid out in Article 8(4) (Schulte in den Bäumen et al. 2010). The authors agree that open consent cannot fulfil the prerequisites for legitimate consent in the Directive, but disagree that such public interest exceptions are necessarily applicable. In this we rather share the interpretation of Kaye (2004). The arguments laid out in footnote 12 in relation to such public interest exceptions in the Regulation are applicable to equivalent Articles in the Directive.

The obvious question which follows from this is: If open consent is a problem (or at least a disputed practise) under the Directive, then why does it seem to continue unchallenged in certain biobanks? This is a question which deserves further research but is beyond the scope of the legal analysis conducted in this article.

^b^This article takes the Regulation as its unit of analysis. The authors feel this is legitimate as the Regulation will constitute a stand-alone piece of legislation. First, this is true in terms of legislative content. Whilst the Regulation emerges from, and shares many similarities with, its predecessor the Directive, the two are not the same law. What was, and is, true in the case of the Directive, will not necessarily be so in the case of the Regulation. Second, this is also true in relation to the Regulation’s potential role in legislating for biobanks. The Directive is applicable to biobanking, but as a legal framework it suffers from a number of flaws making it fragmented, confusing and difficult to apply; differences in national transposition – meaning it lacks harmonisation and cross border biobanking may be subject to numerous regional differences – uncertainty as to how and when it formally applies – does it apply to samples and data, what exactly is the status of genetic data etc. – uncertainty as to which role it plays in the broader body of law applicable to biobanking – with which it sometimes even conflicts. Accordingly, data protection law has been somewhat perceived as a confusing obstruction to the work of biobanks. The Regulation moves toward a resolution of these issues as it will clarify and harmonise data protection law across Europe and provide a European wide interpretation mechanism. Indeed, it may also change the perception of data protection law from a framework of obstruction, to one offering opportunity. As biobanking grows, becomes more networked, more internationalised and comes to occupy a greater role in medical research, there are corresponding increases in calls to clarify the applicable legal provisions. The European level framework of hard law provided by the Regulation would offer one such clear and harmonised legal framework.

^c^A significant portion of modern medical research follows the Genome Wide Association Study methodology. Such studies draw wide scale comparisons between populations with, and without, a certain trait (disease/health condition)

^d^Presumed consent/opt out is often discussed as a form of consent and accordingly, the authors have included it in this section. However, the authors do not see this as a bona fide form of consent. Consent requires an active expression of will to allow another to engage in a practise which would otherwise infringe upon an individual’s rights. In an opt out procedure, no active expression of will is present. Rather, the authors see this as a public interest justification with a secondary deference to individual will.

^e^The authors find the idea of dynamic consent very interesting and very promising. However, it has, up to now, been little used. Equally, a concern which led to inception of open consent was that, with constantly asking research participants for consent to each and every newly proposed research project, research participants would suffer consent overload and would cease to engage with biobanks. Perhaps this risk is also present with dynamic consent.

^f^Sectoral consent may be very useful for limited purpose, or disease specific, biobanks. However, the larger, more prospective and more networked a biobank becomes, the more likely it is that sectoral consent will be regarded as too limited.

^g^However, it should also be noted that the term ‘blanket consent’ appears elsewhere in data protection jurisprudence to refer to a consent with no boundaries at all. This should not be confused with the concept of open consent as we use it in this article – restricted to research uses of collected materials.

^h^Material will only be released to researchers following the consideration of their proposals by mechanisms for quality, and ethics control – Research Ethics Committees, for example, ensure that research proposals fulfil relevant criteria. Samples and data will then be released to the researcher/project under specific agreed conditions of use. It may be the case that information extracted from samples, or the samples themselves are thus further distributed – for example through publication – by the researchers given permission to use the samples.

^i^The technological background to the drafting of the Directive has changed. Developments in the ability to extract and interpret genetic data can be counted among such changes. Simultaneously, the legal context in which the Directive was embedded has changed. The Directive is no longer seen to reflect the European legal architecture of which it forms a part. The signing into force of the Lisbon treaty represented a key moment in the constitutionalisation of data protection law. Two aspects of Lisbon are of particular significance. First, Article 16 of the Treaty of the Function of the European Union (under the heading of ‘general provisions’ rather than ‘internal market’) provides the Union with an explicit legal basis for the adoption of data protection rules. Second, Article 8 of the Charter specifically lists data protection as a fundamental right and Lisbon elevates the Charter to the highest status of EU law. Finally, the Directive has been seen to have serious shortcomings in the harmonization of European data protection law. National transpositions differ significantly – the transposition of the conditions of consent differ considerably from country to country.

^j^The Parliament’s version adds that the application of the Regulation follows ‘***irrespective of the method of processing***’, whilst the Council propose no changes. This does not constitute a substantially significant alteration for the purposes of this analysis.

^k^Recital 23 states: ‘The principles of data protection should not apply to data rendered anonymous in such a way that the data subject is no longer identifiable’ (European Commission 2012a). Although the Parliament’s version - ‘The principles of data protection should ***therefore*** not apply to ***anonymous*** data***, which is information that does not relate to an identified or identifiable natural person. This Regulation does therefore not concern the processing of such anonymous data, including for statistical and research purposes***‘ – and the Council’s version – adding to the Parliament’s ammendments that: ‘***The principles of data protection should not apply to deceased persons’*** – contain alterations to the Commission’s text, these do not serve to change the substantial meaning of the Article for the purposes of the current analysis. Many large biobanks subject samples and data to procedures aimed at disguising the identity of the original data subject – pseudonymising data. The information allowing the subject to be re-identified is still retained within the biobank, but is not passed to third parties (for example research projects). It is clear that pseudonymous data are not anonymous and do fall under the scope of data protection law. The Article 29 Working Party clearly state that: ‘retraceably pseudonymised data may be considered as information on individuals which are indirectly identifiable’ – indirect identifiability qualifies data as ‘personal’ within the meaning of data protection law (Article 29 Data Protection Working Party 2007a, p.18). The Parliament clarified this by clarifying pseudonymous data as falling within the scope of protection – although they regard the severity of consequence as much lower than with the processing of regular personal data. Biobanks may also claim to process truly anonymised data. However, there are serious doubts about the effectiveness and applicability of either anonymity, or pseudonymity procedures to genetic data – the authors have discussed this elsewhere (Hallinan et al. 2013). Equally, the ever expanding quantity of data which biobanks will collect and generate about a data subject means it is difficult to state that any anonymisation or pseudonymisation process will retain efficacy over time. Accordingly, the authors do not do not believe that data in biobanks should be regarded as effectively anonymised or pseudonymised.

^l^Data protection is aimed at regulating the processing of personal information. Considered narrowly, samples are not information, therefore cannot be ‘personal data’ and therefore cannot fall under the scope of protection. Samples are only valuable however, as they contain data – it is simply that this data requires further processing to be converted into readable form. Accordingly, they seem to blur the clear line between informational and corporeal. The Regulation offers no direct answer to this question, and the available legal sources on both national and international level differ in their interpretation. For example, on the one hand, the Article 29 Working Party offer guidance to the effect that samples cannot be considered as data (Article 29 Data Protection Working Party 2007a, p. 9). On the other hand, the European Court of Human Rights suggests they can (S. and Marper v United Kingdom, European Court of Human Rights 2008, § 68). In this situation of uncertainty, the authors feel that the interpretation of Bygrave is correct, and the Regulation should be found to apply to samples (Bygrave 2010). Given that the collection and storage of samples constitutes a key part of the chain of processing, and the Regulation has been tasked with protecting individuals’ interests when their data are being processed, it would make logical and practical sense to apply data protection law to biological samples in the biobanking context.

^m^The Parliament have added ‘***philosophical*** beliefs…***sexual orientation or gender identity,*** trade union…***activities***…***biometric*** [data and data on] ***administrative sanctions, judgments,*** criminal ***or suspected offences’*** to this list. The Council have further suggested that ‘race’ in the Commission’s version, be changed to ‘***racial***’. These changes are of some significance, but do not change the substantial relevance of the Article for the purposes of this analysis.

^n^The authors have issues with both proposed approaches to the definition of ‘genetic data’. On the one hand, the Commission’s definition is very broad. There is almost no personal data which does not relate to some inherited characteristic. It is clearly not the case that all personal data should be classified as ‘genetic’. Accordingly, the Commission’s definition of genetic data is likely to be far too broad and would require considerable interpretation to narrow its scope. On the other hand, the definition offered in the Parliament and Council versions removes the possibility to interpret any data, other than that which has been revealed through a specific type of analysis, as genetic. This would mean that all data which could be conceived of as being genetic in content, but which was not revealed as the result of a genetic analysis, would not be regarded as ‘genetic data’. Information as to whether an individual suffered from haemophilia, for example, would be regarded as genetic if produced through analysis of the individual’s genome, but would not be regarded as ‘genetic’ if revealed as a result of an alternative means of diagnosis. Why should it be the process of revealing the information which makes the information itself ‘genetic’ or not. Further, it excludes all types of information which could be subject to a genetic interpretation – i.e. could be used to reveal genetic information in the future. For example, under this definition, biological samples would not be conceived of as ‘genetic’, because they had not yet been subject to a ‘genetic analysis’. This is problematic for at least two reasons. First, in relation to biological samples, the authors have already expressed their opinion – elaborated in footnote 14 – that, in the biobanking context, the Regulation should apply to biological samples. Moving from this interpretation, it is only logical that samples be considered as ‘genetic data’. Given that such samples are only collected for the genetic information they contain, conceiving of them as any other type of data seems absurd. Second, in *S v. Marper* the European Court of Human Rights considered the sensitivity of genetic data when considering the right of the police in the UK to retain genetic samples (S. and Marper v United Kingdom, European Court of Human Rights 2008). They concluded that such data might be considered as ‘sensitive’ because it had the future potential to reveal a great deal of information, some of which – for example health information – would also be regarded as sensitive. The Article 29 Working Party also considered the future possibilities of interpretation as a significant factor in the sensitivity of genetic data (Article 29 Data Protection Working Party 2004a). In the case of the biological sample, all possibilities for future revelation exist before any analysis has been conducted. The exclusion of the possibility to consider data as genetic through content, or interpretative potential, or even context of interpretation, is very restrictive. Whilst the focus solely on analysis is useful in narrowing down a definition, it leads to some arbitrary results both in relation to which data is seen as ‘genetic’ and why this data should be seen as ‘sensitive’, whereas other forms of data, which display the same characteristics, are excluded.

There remains a lack of clarity regarding the conceptualisation of genetic data in data protection law. Accordingly whichever interpretation is adopted, it is unlikely that it will be regarded as iron-clad and the authors believe considerable interpretation will be necessary.

^o^The clause on the prohibition on processing sensitive data remains unchanged in the Parliament and Council versions. The Parliament adds two further exceptions under 9(2): 9(2)(aa) relating to the excecution of a contract and 9(2)(ja) relating to processing for archiving purposes. The Council have suggested the deletion of Article 9(2)(j) – relating to the processing of data relating to criminal convictions. Neither of these amendments are relevant for the current analysis.

^p^The Parliament and Council versions retain consent as a justification to process sensitive data in 9(2)(a).

^q^There is a small nuance to this argument which is elaborated in the first paragraph of footnote 21.

^r^There are also practical reasons consent ought to be sought. The scope and success of medical research relies on the involvement, and trust, of research participants. In biobanking, fostering such trust is tightly linked to the involvement of research subjects in the biobanking process. The consent process puts the individual in charge of their own participation, and accordingly over their input into the biobanking project.

^s^Whilst the authors adhere to the position that there is a lexical order to legitimating grounds under Article 9(2), it has been argued that this is not the case. Equally, it has been suggested that – as will be argued in the following sections of this article – if open consent cannot be regarded as legitimate under the conditions of consent in the Regulation, this means that biobanks are in fact *not* able to obtain consent as understood in 9(2)(a). The authors also object to this position. Open consent is the most convenient and research-optimal consent mode for biobanks as it allows maximum possible research use of samples with minimum administrative burden. However, this does not mean that biobanks’ work is necessarily impossible under the conditions laid out in 9(2)(a). Certainly, this mean administrative inconvenience and less optimal opportunities to use samples but this is a different proposition to the suggestion that biobanks could *not* obtain consent as understood under 9(2)(a). This is especially true given consent modes made possible through technology – see above, for example, on dynamic consent.

Nevertheless, if either of these arguments are taken forward, a different Article 9(2) exception would need to be found under which biobank processing could be legitimated. There are only a limited number of possible exemptions among 9(2)(b) – (j) which seem applicable. In particular, 9(2)(g) and (9)(2)(i) have been put forward (Schulte in den Bäumen et al. 2010 pp, 37–38). Further inspection of these grounds shows 9(2)(i) as offering a real possibility to legitimate biobank processing, but only in the Commission and Council versions.

1 Article 9(2)(g): This allows processing of sensitive data if ‘processing is necessary for the performance of a task carried out for reasons of high public interest, on the basis of Union law, or Member State law which shall be proportionate to the aim pursued, respect the essence of the right to data protection and provide for suitable measures to safeguard the fundamental rights and the interests of the data subject’. There are at least four objections which could be made to the applicability of 9(2)(g). First, there is no encompassing European level legislation on biobanking. Accordingly, any justification on the grounds of 9(2)(g) would need to be on the basis of a relevant national provision. National provisions would likely be divergent. In legal terms, this is undesirable as it would mean a fragmentation of data protection law relating to biobank research. In practical terms, a key obstacle which has hampered international biobanking practise has been the lack of a harmonised legal framework. The Regulation would thus be a golden opportunity for biobanking – one which would be missed if justification for processing were to be found in national law. Second, use of Article 9(2)(g) would require biobanking research to qualify as a ‘high public interest’. Whilst there is no clarification as to exactly what constitutes a ‘high public interest’, one would presume that research in the ‘high public interest’ would need to be focussed on producing imminent output to meet a specific pressing social need. It is debatable whether biobanks do this (Rouvroy 2008, pp 13–55). The value of large scale biobank based research in terms of usable medicines (at least in relation to the amount of resourced invested) has yet to materialise. Third, it is not always the case that biobanks are operated purely in the public interest. The Icelandic DeCode bank, for example, was owned by a private corporation (Arnason 2007, pp 1–2). Nor is it certain that the results will be directly for public benefit. Application to biobank resources may be open to private institutions. First line beneficiaries of research would then be company shareholders, rather than the public at large. Equally, the beneficiary ‘public’ may be quite different to the public in whose name biobanking was supposedly a ‘high interest’. For example, research done on biobank samples sent abroad may eventually be of benefit in another country. Finally, in order for 9(2)(g) to apply, there must be national legislation which provides for ‘suitable measures to safeguard the fundamental rights and the interests of the data subject’. Biobanking poses a specific set of risks to the data subject. This is especially due to the ever expanding quantity of information they collect, the fact that genetic data is collected and the fact that the uses of this data are uncertain at the moment of collection. Accordingly, reliance on 9(2)(g) would arguably need to be on the back of a specific law reflecting the specifics of biobanking research. General legislation – for example legislation providing a general exemption for research purposes – would fail to ‘safeguard the fundamental rights and the interests of the data subject’. Such biobank specific legislation exists in only few Member States.

2 Article 9(2)(i): This allows processing of sensitive data if ‘processing is necessary for historical, statistical or scientific research purposes subject to the conditions and safeguards referred to in Article 83’. In Article 83 of the Commission and Council versions, biobanks could certainly fulfil the obligations laid out. If the primacy of consent is questioned, or it is seen that it is impossible for the biobank to gain consent under the conditions of 9(2)(a), then these versions of Article 83 would provide a viable alternative for legitimating biobank processing. However, the conditions of Article 83 are the subject of some disagreement. When health data are processed under 9(2)(i) of the Parliament’s version, Article 83 must be read in conjunction with Article 81. This Article applies to biobanks. Biobanks may process data which can be directly identified as ‘health data’ in the form of medical records. However, Recital 26 also clarifies that ‘health data’ include both ‘information derived from the testing or examination of a body part or bodily substance, including biological samples; or any information on e.g. a disease, disability, disease risk, medical history, clinical treatment, or the actual physiological or biomedical state of the data subject’. Accordingly, almost all biobank processing can also be conceived of as the processing of health data. Article 81 contains three clauses which seem of significance: Article 81(1b) and Article 81(2) and Article 81(2a). Article 81(1b) states that: ‘Where the data subject's consent is required for the processing of medical data exclusively for public health purposes of scientific research, the consent may be given for one or more specific and similar researches. However, the data subject may withdraw the consent at any time’. Anecdotally, the authors have heard that this provision was meant to cover the possibility for open consent to be used in scientific research involving health data. However, we have not found any clear indication that this is the case. It is true that Article 81(1b) would allow the data subject to give consent to multiple research uses in advance. However, the authors feel that Article 81(1b) does not practically open up any new possibilities which would not be possible under a justification following 9(2)(a). A data subject is quite free to give any number of specific consents to different processing operations. Simply because these are secured at the same time and place does not, of itself, serve to invalidate any, or all of them. By extension, the authors believe that this provision, by itself, cannot serve to legitimate open consent. Under 81(1b) each research use would still need to be specifically elaborated. In turn, the scope of this ‘collection of consents’ is limited to research which is ‘similar’ – although quite how broadly this could be interpreted is unclear. This is still a significant step short of open consent, which constitutes one consent, for all future research purposes. In open consent, no research purpose is specifically clarified, nor is it necessarily the case that research purposes will be ‘similar’ to one another. Article 81(2) states that health data may only be processed for research ‘with the consent of the data subject’. In Article 81, there are no further clarifications to the concept of ‘consent’. There is no reference to any special conditions under which Article 81 consent should be regarded as legitimate, nor are there any indications that Article 81 consent refers to a concept of consent from another area of law. Therefore we must conclude that ‘consent’ in Article 81 must refer to ‘consent’ as it appears elsewhere in the Regulation and is subject to the same conditions. Finally, Article 81(2a) provides an exception to consent for medical research, this follows only for ‘research that serves a high public interest if that research cannot possibly be carried out otherwise’. As we have argued earlier in this footnote, we find it difficult to automatically justify biobanking as research which serves a ‘high public interest’.

^t^There are a number of other conditions which must also be fulfilled. Certain of these may also cause problems for open consent. However, the requirement that consent be ‘specific and informed’ is perhaps the biggest obstacle to open consent in data protection law. The Parliament’s version retains the same wording as the Commission’s. The Council alters the wording slightly, removing the term ‘explicit’. The Council add that consent must be ‘explicit’ in 9(2)(a) of their version. The concept of ‘explicit’ particularly refers to the signalling requirements of a given consent, rather than the informational content of that consent.

^u^This may not always be the case – for example in the case of children or agent given consent – but for the purpose of this article we will remain with the generally applicable situation.

There are a number of changes to the Commission text in the Parliament and Council versions. Only those of significance will be specifically listed.

^v^In their version of Article 14, the Council have also removed the obligation for the controller to inform the data subject as to how long information will be stored for. They also have not seen it necessary to include information corresponding with the Commission‘s version Article 14(gb) and 14(ha). None of these alterations is relevant for the current analysis.

^w^Whilst this is not explicitly mentioned in Article 14, it could be seen to be implied in the Regulation. First, ‘the data’ are referred to a number of times in relation to the other categories of information. Second, the aim of Article 14 is to provide the data subject with information describing the relevant factors of a proposed processing operation. It would be almost impossible for the data subject to begin to conceive of facts and implications of a processing operation without information as to ‘what’ is actually being processed. It may be that the legislator presumed that it was obvious that, when data are collected from a data subject, that this subject would know what ‘data’ they were.

^x^Neither Article 14(1), nor the explanations of the Article 29 Working Party create an exhaustive list of categories of data comprising the informational obligations of the data controller. Article 14(1) explicitly states the necessity to provide the data subject with; ‘any further information necessary to guarantee fair processing’ (European Commission 2012a). Whilst it is not easy to draw categories of information directly from the idea of ‘fair processing’, it is possible to generally link the idea of fair processing to risk in processing. Indeed, the Parliament’s version of 14(h) extends this obligation to include information relating to: ‘***the existence of certain processing activities and operations for which a personal data impact assessment has indicated that there may be a high risk’.*** Accordingly, it can be argued that any aspect of processing, relevant to defining the risk which might arise from processing, could fall under the category of ‘additional information necessary’ – depending on the context. In the case of biobank based research – which would be subject to an impact assessment under the conditions laid out in Article 33 – the processing of samples and data is done through the framework provided by genetic science. This interpretative framework has rules and limits. These rules and limits define what can and cannot be done with samples and materials. Accordingly, they shape the implications (and risk) of processing for the data subject. With this in mind, the authors believe that certain information as to the characteristics of the interpretative framework ought to be communicated to the data subject. This would constitute a separate category in itself. We have left this out of the paper, as there are no current statutory, or jurisprudential guides as to the necessity of this information. Accordingly, there is no evidence that this category should be a legal problem for the legitimacy of open consent.

However, the authors do wonder how much information would need to be provided under this ground, and whether this could be provided within a consent procedure – genetic science is complicated and vast.

At first glance, the answer appears to be yes. The obligation that the data subject understands some features of genetic science is not limited to biobanks employing open consent. It is applicable to all situations in which genetic data are processed. Article 9 of the Regulation lists ‘genetic data’ as a form of data which can be processed with the data subject’s consent. As the Regulation confirms the normative position that the data subject can consent to the processing of their genetic data it also implicitly suggests that the controller must be able to communicate the relevant information about genetic science. Following this line of reasoning, the obligation to communicate some amount of genetic science to the data subject does not, in principle, constitute a legislative block to open consent. It should be noted however, that this does not mean that practically this is not an issue. Current open consent procedures in Europe do not appear to even attempt to explain any aspect of the interpretative framework which will be applied to data collected. In the consent materials for both the UK biobank and the Geenivaramu – the Estonian biobank – there is not a single reference to the basics of genetics.

At a second glance however, it seems a counter-argument could also be put forward. In the history of data protection law, there have been a number of approaches which have sought to limit the ability of, and the situations in which, the data subject could consent. One line of argumentation – which has been increasingly prominent as technology has developed – is that there are situations in which the background processing operations are significant to the consequences of the processing but are too complex for the data subject to understand (Solove 2012). In ‘The Future of Privacy’ – an early contribution to the reform process leading to the Regulation – the Article 29 Working Party summarize this argument: ‘the requirement that consent has to be informed starts from the assumption that it needs to be fully understandable to the data subject what will happen if he decides to consent to the processing of his data. However, the complexity of data collection practices, business models, vendor relationships and technological applications in many cases outstrips the individual’s ability or willingness to make decisions to control the use and sharing of information through active choice’ (Article 29 Data Protection Working Party 2009). This line of argumentation has, up to now, been focused at processing in the online environment. However, the authors see that a direct comparison could be made between the complexity of ICT and the complexity of genetics. Indeed, the concern that ‘average’ citizens might face a knowledge deficit relating to genetics was taken seriously by the Personal Genome Project – a US/Canadian genetics project employing open consent procedures. In the initial round of recruitment, only those with at least a master’s degree in genetics were allowed to consent to participate in the project (Lunshof et al. 2008).

^y^They do begin by providing a generally applicable rule (although the threshold set by this is absurdly low): ‘blanket consent without specifying the exact purpose of the processing is not acceptable’ (Article 29 Data Protection Working Party 2011, p 17).

^z^However, it should be noted that consent under the Regulation fails if information in only one category cannot be provided, or is not adequately specific.

^aa^Article 14(b) in the Parliament’s version and Article 14(1)(b) in the Council’s version. It should be pointed out that the Parliament’s version of Article 9(2)(a) specifically includes the possibility to consent to ‘one or more specified purposes’ or processing. However, as we explain in footnote 21, we do not see this as opening up a route to broadening the scope of a single consent, but rather to allowing multiple, specific consents to be given at one time. Open consent does not attempt to list multiple specific uses.

^bb^The purpose limitation principle is retained in Article 5 in both the Parliament and Council versions.

Article 83c(3) of the Council version should also be noted here. ‘Article 83c(3) states: By derogation from points (b) and (e) of Article 5(1) and from Article 6(3a), processing of personal data for scientific (…) purposes under the conditions referred to in paragraph 1 shall not be considered incompatible with the purpose for which the data are initially collected and may be processed for those purposes for longer than necessary for the initial purpose, provided that the controller implements appropriate safeguards for the rights and freedoms of data subjects, in particular (…)’. The sentiments of Article 83c(3) mirror those of Article 5(b) of the Council’s version which states that; ‘further processing of data for historical, statistical or scientific purposes shall not be considered as incompatible subject to the conditions and safeguards referred to in Article 83. At first sight, this looks like a possibility for biobanks to avoid specificity as long as they institute adequate safeguards. However, the Article only applies to data whose initial collection was not for the purposes of scientific research. Such data may then be repurposed for a secondary use in scientific research provided adequate safeguards are in place. In biobanks operating open consent, data are collected with the primary purpose being scientific research. The biobank is fully aware, at the moment of collection, of the scope of research which may be conducted – although they cannot be specific about what research exactly. There is no secondary purpose. A biobank cannot thus rely on this Article to avoid the limitations of consent provisions in the Regulation. There is however, irony in the fact that data collected without the primary purpose being research, can be used for a much broader set of research purposes than data initially collected for research with the data subject’s consent.

^cc^Article 14(f) of the Parliament’s version and Article 14(1a)(c) of the Council’s version.

^dd^At first sight, the issue raised by stating ‘researchers’ as ‘recipients’ seems very similar to that raised by stating ‘research’ as ‘purpose’. Accordingly, a similar line of questioning would seem relevant. However, there is a difference in the construction of the Articles which deserves consideration. Article 14(1)(f) states that the data controller must list ‘the recipients *or* the categories of recipients’ of the data. 14(1)(b) on the other hand, makes no reference to the possibility to elaborate ‘categories’ of purpose. Unfortunately, it has proven difficult to clarify the relevance of this difference. There is no clear indication as to the purpose of the inclusion of the term ‘categories’ in relation to recipients of data. The authors feel the most likely interpretation of the term is that it was inserted in order to give some freedom from the narrow term ‘recipients’ which appears at the start of Article 14(b). If, ‘categories of recipient’, were not there, the data controller would always be obliged to provide an exact list of all recipients, at the moment of consent – similar to the strict construction we find in Article 14(g). Given that consent is a highly contextual concept, the authors cannot imagine the benefit to be had from setting such a condition. Another interpretation of the term is possible. It could be interpreted as a qualification. The term ‘categories of recipient’ allowing a looser degree of specificity in relation to information on recipients in comparison with other categories of information in Article 14 (for example purpose in 14(b)). If this interpretation is correct, it would suggest that the Commission wanted to create a comparison of categories of information. The authors find this idea unlikely.

^ee^Article 14(g) of the Parliament’s version and Article 14(1a)(d) of the Council’s version.

^ff^Some of this data may be collected to facilitate administration. Other forms of data, such as lifestyle data, may be collected in order to aid research. Such lifestyle information allows studies to be conducted that observe the interplay of genetics and environmental factors – for example, gene X plays a role in condition A, but so may smoking: the complete causes of condition A would thus require information on study participant smoking habits as well as their genetics.

^gg^Although each individual’s specific genetic make-up is unique, specific architectural features will rarely be unique to one individual. Accordingly, if a feature of architecture has significance, it can often be generalised to infer knowledge about others who may be assumed to share that architecture (for example blood relatives – including ancestors and future progeny) but a number of other shared biological groups could be imagined). Genetic data may thus also have relevance for successive generations.

^hh^The ability of technological progression to invalidate claims made at a certain moment of time has been recognised in data protection law in relation to anonymous data. The relation of technological progression to consent, however, remains unclear (Article 29 Data Protection Working Party 2007a).

^ii^It has been pointed out that there may be the facility to exclude the applicability of Article 14, for example, in Article 83c of the Council’s proposal on Chapter IX. However, this Article only becomes relevant when data are processed on the basis of 9(2)(i) – processing of sensitive data for scientific purposes – not when the processing of data is legitimated under 9(2)(a) (consent). We have argued that we believe there is a lexical order to grounds of legitimation and that 9(2)(a) should be relied upon. Further Article 83c(2) states: ‘Where personal data are processed for scientific purposes, Member State law may, subject to appropriate measures to safeguard the rights and freedoms of the data subject, provide for derogations from: (a) Article 14a(1) and (2) where and insofar as the provision of such information proves impossible or would involve a disproportionate effort.’ As elaborated in footnote, 21, such legislation either does not currently exist or may be inadequate and, if relied on, would perhaps be counterproductive as it may lead to a fragmentation of the legislative framework.

^jj^It should be borne in mind that other actors may become involved later in the biobanking process – pharmaceuticals companies who want to create products on the back of biobank materials, police forces who want to use biobank materials for crime detection etc. However, these relationships raise issues in their own right and there may be the need to provide legal protection against the harms that could come through the use of data by third parties whose interests may indeed conflict with the donor’s. However, the authors are sceptical that strict consent requirements in data protection law are the best place to do this. In relation to police use of data, for example, strict consent will be no protection at all. Statutory biobank secrecy provisions would be far better suited.
